# Excess Mortality Among Patients in the Veterans Affairs Health System Compared With the Overall US Population During the First Year of the COVID-19 Pandemic

**DOI:** 10.1001/jamanetworkopen.2023.12140

**Published:** 2023-05-08

**Authors:** Daniel M. Weinberger, Liam Rose, Christopher Rentsch, Steven M. Asch, Jesse A. Columbo, Joseph King, Caroline Korves, Brian P. Lucas, Cynthia Taub, Yinong Young-Xu, Anita Vashi, Louise Davies, Amy C. Justice

**Affiliations:** 1 Department of Epidemiology of Microbial Diseases, Yale School of Public Health, New Haven, Connecticut; 2 Department of Veterans Affairs Connecticut Healthcare System, West Haven; 3 Department of Veterans Affairs Medical Center, Palo Alto, California; 4 Surgery Policy Improvement Research and Education Center, Stanford School of Medicine, Palo Alto, California; 5 Department of Non-communicable Disease Epidemiology, London School of Hygiene & Tropical Medicine, London, United Kingdom; 6 Department of General Internal Medicine, Yale School of Medicine, New Haven, Connecticut; 7 Division of Primary Care and Population Health, Stanford School of Medicine, Palo Alto, California; 8 Department of Health Research and Policy, Stanford School of Medicine, Palo Alto, California; 9 Department of Veterans Affairs Medical Center, White River Junction, Vermont; 10 Department of Medicine, Geisel School of Medicine at Dartmouth, Hanover, New Hampshire; 11 Neurosurgery, Yale School of Medicine, New Haven, Connecticut; 12 Dartmouth Hitchcock Medical Center, Lebanon, New Hampshire; 13 Department of Emergency Medicine, University of California, San Francisco; 14 Department of Surgery, Geisel School of Medicine at Dartmouth, Hanover, New Hampshire; 15 Department of Health Policy and Management, Yale School of Public Health, New Haven, Connecticut

## Abstract

**Importance:**

During the first year of the COVID-19 pandemic, there was a substantial increase in the rate of death in the United States. It is unclear whether those who had access to comprehensive medical care through the Department of Veterans Affairs (VA) health care system had different death rates compared with the overall US population.

**Objective:**

To quantify and compare the increase in death rates during the first year of the COVID-19 pandemic between individuals who received comprehensive medical care through the VA health care system and those in the general US population.

**Design, Setting, and Participants:**

This cohort study compared 10.9 million enrollees in the VA, including 6.8 million active users of VA health care (those with a visit in the last 2 years), with the general population of the US, with deaths occurring from January 1, 2014, to December 31, 2020. Statistical analysis was conducted from May 17, 2021, to March 15, 2023.

**Main Outcomes and Measures:**

Changes in rates of death from any cause during the COVID-19 pandemic in 2020 compared with previous years. Changes in all-cause death rates by quarter were stratified by age, sex, race and ethnicity, and region, based on individual-level data. Multilevel regression models were fit in a bayesian setting. Standardized rates were used for comparison between populations.

**Results:**

There were 10.9 million enrollees in the VA health care system and 6.8 million active users. The demographic characteristics of the VA populations were predominantly male (>85% in the VA health care system vs 49% in the general US population), older (mean [SD], 61.0 [18.2] years in the VA health care system vs 39.0 [23.1] years in the US population), and had a larger proportion of patients who were White (73% in the VA health care system vs 61% in the US population) or Black (17% in the VA health care system vs 13% in the US population). Increases in death rates were apparent across all of the adult age groups (≥25 years) in both the VA populations and the general US population. Across all of 2020, the relative increase in death rates compared with expected values was similar for VA enrollees (risk ratio [RR], 1.20 [95% CI, 1.14-1.29]), VA active users (RR, 1.19 [95% CI, 1.14-1.26]), and the general US population (RR, 1.20 [95% CI, 1.17-1.22]). Because the prepandemic standardized mortality rates were higher in the VA populations prior to the pandemic, the absolute rates of excess mortality were higher in the VA populations.

**Conclusions and Relevance:**

In this cohort study, a comparison of excess deaths between populations suggests that active users of the VA health system had similar relative increases in mortality compared with the general US population during the first 10 months of the COVID-19 pandemic.

## Key Points

**Question:**

How did changes in rates of death among patients in the Veterans Affairs (VA) health system that occurred during the first year of the COVID-19 pandemic compare with changes in rates of death among the general US population?

**Findings:**

This cohort study found that changes in death rates between the VA population and the general US population were comparable after accounting for demographic differences between the populations. The changes varied by race and ethnicity in both populations; these disparities changed over time.

**Meaning:**

This study suggests that the COVID-19 pandemic had a large association with rates of death in the US and, specifically, among veterans.

## Teaser

This cohort study quantifies and compares the increase in death rates during the first year of the COVID-19 pandemic betweeen individuals who received comprehensive medical care through the Veterans Affairs health care system and those in the general US population.

## Introduction

During the first year of the COVID-19 pandemic, there was a substantial increase in the rate of death in the United States.^1^ This increase, particularly among older adults, was largely a consequence of infection with SARS-CoV-2.^2^ However, the association of the pandemic with death rates varied substantially across subpopulations. Rates of death related to COVID-19 were higher among Black and Hispanic populations compared with White populations, among men compared with women, and among older adults compared with younger individuals.^3^ These disparities were associated with many factors, including social determinants of health (eg, employment type and household composition), differing comorbidities, systemic differences in access to health care, and biological differences in immune responses by age and sex.

Many evaluations of the impact of COVID-19 have taken advantage of data from the US Department of Veterans Affairs (VA), which provides comprehensive care nationally for veterans of the armed forces. Due to the unique demographic characteristics of this population, questions have been raised about the relevance of studies in this population. Previous reports show that death rates among those who typically received care in the VA health system were less affected than death rates among the general US population during the COVID-19 pandemic, despite higher rates of comorbidities in the veteran population.^4,5,6^ However, veterans enrolled in VA health care also differ from the general US population in age, sex, racial and ethnic composition, and geographic distribution, and each of these factors has been associated with mortality rates in general and death rates due to COVID-19 specifically.^7,8,9^ Using detailed data on individual deaths, it is now possible to make appropriate comparisons by accounting for the differing demographic characteristics of veterans receiving care from the VA health system vs individuals in the general US population. The aim of this analysis was to quantify excess all-cause deaths during the first 9 months of the COVID-19 pandemic among veterans enrolled in the VA health system compared with the general US population and to obtain standardized overall estimates of excess deaths, adjusted for age, sex, race and ethnicity, and geographic region.

## Methods

### Overview

The analyses focused on deaths among adults aged 25 years or older in 3 different populations: VA health system enrollees, VA health system active users, and the general US population. Data were obtained from the National Center for Health Statistics (NCHS) and from the VA health system for deaths occurring between January 1, 2014, and December 31, 2020. Time series were created by counting the number of deaths by age group, sex, race and ethnicity, year, quarter, and Census region (West, South, Midwest, and Northeast). A model was fit to quarterly data from 2014 to 2019 (before the pandemic) and then extrapolated to 2020. Excess deaths and risk ratios (RRs) were calculated by comparing the observed number of deaths with the expected number of deaths. This study was reviewed and approved by the institutional review boards at the Department of Veterans Affairs West Haven, Palo Alto, and White River Junction. Patient informed consent was not applicable because this study is a secondary analysis of mortality data. This study conforms to the relevant sections of the Strengthening the Reporting of Observational Studies in Epidemiology (STROBE) reporting guideline for cohort studies.

### Data Sources and Definitions

#### National Center for Health Statistics

Individual-level data representing all deaths in the US were obtained from the NCHS through a data use agreement that allowed for the sharing of the geographic locations of the deaths.^10^ The individual-level data, except for geographic region, are publicly available and can be downloaded from the NCHS website.^10^ These vital statistics data have detailed information on the deceased individual, including age (years), sex, race and ethnicity, state and county of residence, and the underlying and contributing causes of death (coded using the *International Statistical Classification of Diseases and Related Health Problems, Tenth Revision*).

Population size data for the general US population, collected by the US Census Bureau, were obtained from the bridged race files (Vintage 2020) produced by the NCHS. These data are available at the annual scale (July 1 estimates). To estimate population size for each stratum by quarter, we used linear interpolation.

#### Department of Veterans Affairs

The population of VA enrollees includes approximately 10.9 million individuals enrolled in VA health care: 9.2 million veterans of the US armed forces and 1.7 million who are family members of disabled veterans.^11^ Most enrollees have another form of health insurance, with Medicare being the most common among those aged 65 years or older.^12^ The baseline comorbidities and rates of deaths varied between these populations, and as such, we included 2 definitions in our analysis: “VA enrollees” and “VA active users”.

The population of VA active users were individuals with at least 1 diagnosis in their VA health system electronic health record in the 2 years prior to each time point, indicating at least 1 clinical encounter within the VA health system.^13^ The VA active user cohort is dynamic, with the population determined by activity in the previous 2 years from each time point. Population size data for the VA populations were obtained by querying the Assistant Deputy Under Secretary for Health Enrollment Files for each fiscal year.

#### Definitions

These analyses focused on deaths among adults aged 25 years or older. The 3 populations are nested within each other (VA active users are also VA enrollees; VA enrollees are also part of the general US population). The analyses focused on deaths among adults aged 25 years or older because there are very few veterans younger than 25 years (1.3% of veterans).

For each population, we used the same definitions for creating subgroups:

1. Age group (25-44, 45-64, 65-74, 75-84, and ≥85 years).
2. Sex (male or female).
3. Race and ethnicity: American Indian or Alaska Native (non-Hispanic); Asian, Native Hawaiian, or Other Pacific Islander (non-Hispanic); Black or African American (non-Hispanic); Hispanic; and White (non-Hispanic). These groups were chosen to align with population size data compiled by the NCHS.^14^ Race and ethnicity for the VA population were based on self-reported race and ethnicity. Race and ethnicity (single race or bridged race categories) for the NCHS data are based on report from funeral directors, which are provided by an informant, such as next of kin, or based on observation of the funeral director. Recording of some racial and ethnic groups in the NCHS data are less accurate, particularly for American Indian or Alaska Native individuals.^15^ For both data sets, individuals who reported Hispanic ethnicity were included in the Hispanic group, regardless of any other self-reported race.
4. Quarter (January to March, April to June, July to September, and October to December).
5. Census region (Northeast, South, West, and Midwest).^16^

### Statistical Analysis

#### Model to Generate a Baseline

Statistical analysis was conducted from May 17, 2021, to March 15, 2023. All statistical tests were 2-sided. The goal for the model was to generate an expected number of deaths by month and subgroup based on prepandemic quarterly data from the preceding 6 prepandemic years (2014-2019) and then extrapolate to each quarter in 2020. Because the pandemic activity increased in the US in March 2020, the first quarter of 2020 includes both pandemic and prepandemic months and is therefore not used for model fitting. Excess deaths and RRs are calculated by comparing the observed number of deaths with the expected number of deaths based on the model. Observed and modeled values were combined over subgroups to obtain summary estimates. We used a multilevel regression model fit to data aggregated by time period, race and ethnicity, age group, region, and sex. Models were fit in a bayesian setting using the INLA package in R, version 4.1.2 (R Group for Statistical Computing) (eMethods in Supplement 1).

#### Standardized Death Rates

For comparisons between the different populations, we used death rates standardized by age, sex, race and ethnicity, and region, using direct standardization. The population of VA enrollees who have nonmissing data on race and ethnicity was used as the standard population. Both the observed and expected death rates were standardized, and the ratio of these standardized values provided a mortality RR adjusted for population structure.

#### Individual-Level Analysis

We used the full patient-level data from VA active users to compare estimates of excess mortality with the Poisson model described. These were fit to aggregated time series data, with a patient-level Cox proportional hazards regression model fit to individual-level data (eMethods in Supplement 1).

#### Comparison With Reported COVID-19 Deaths

For the NCHS data, we compared the estimates of excess deaths due to COVID-19 with deaths recorded as being caused by COVID-19. COVID-19 deaths were identified based on having a code for the underlying or contributing cause of death listed as U07.1, which was the code primarily in use during 2020.

#### Availability of Code and Data

Code used for all analyses can be found online.^17^ All analyses were completed in R, version 4.1.2.^18^ Data on US mortality, with the exception of state or region, can be obtained online.^19^ For additional variables, including geography, a data use agreement with NCHS is required. US Department of Veterans Affairs data and the analytic data sets used for this study can be made available to researchers with a VA institutional review board–approved study protocol and data use agreement. Information is available online^20^ or by contacting the VA Information Resource Center.

## Results

### Demographic Characteristics and Baseline Death Rates

There were 10.9 million enrollees in the VA health care system and 6.8 million active users (Table 1). The demographic characteristics of the VA population differed from those of the general US population in several important ways. The VA population was predominantly male (>85% in the VA health care system vs 49% in the US population), was older than the general US population (mean [SD], 61.0 [18.2] years in the VA health care system vs 39.0 [23.1] years in the US population), and had a larger proportion of individuals who were White (73% in the VA health care system vs 61% in the US population) or Black (17% in the VA health care system vs 13% in the US population) and smaller proportion who were Hispanic (7% in the VA health care system vs 18% in the US population) or Asian, Hawaiian, or Pacific Islander (2% in the VA health care system vs 6% in the US population). Expected death rates were generally higher among the VA enrollees than the general US population (2520 per 100 000 [95% prediction interval, 2360-2670 per 100 000] vs 930 deaths per 100 000 [95% prediction interval, 910-960 per 100 000]) and higher still among active users of VA health care (2910 deaths per 100 000 [95% prediction interval, 2770-3050 per 100 000]) (Table 2). Even after standardizing the death rates by age, sex, race and ethnicity, and region, the expected death rate among the active users of VA health care was approximately 26% higher than in the general US population (2160 per 100 000 [95% prediction interval, 2130-2200 per 100 000] in the US population vs 2730 per 100 000 [95% prediction interval, 2590-2860 per 100 000] among VA active users).

**Table 1.** Characteristics of the Populations in 2019^a^

Characteristic	US population (n = 328 329 953)	VA population	
		Enrollees (n = 10 939 936)	Active users (n = 6 837 558)
Age, mean (SD), y	39.0 (23.1)	61.0 (18.2)	62.2 (17.2)
Age group, No. (%)^b^			
<25 y	103 323 542 (31.5)	195 689 (1.9)	61 186 (1.0)
25-44 y	87 678 128 (26.7)	2 108 249 (20.1)	1 184 766 (18.1)
45-64 y	83 291 548 (25.4)	3 052 556 (29.0)	1 900 716 (29.0)
65-74 y	31 471 344 (9.6)	2 825 582 (26.9)	1 972 179 (30.1)
75-84 y	15 965 924 (4.9)	1 380 960 (13.1)	873 952 (13.3)
≥85 y	6 599 467 (2.0)	946 160 (9.0)	565 774 (8.6)
Race and ethnicity, No. (%)^c^			
American Indian or Alaska Native	2 753 041 (0.8)	61 684 (0.7)	43 446 (0.7)
Asian, Native Hawaiian, or Other Pacific Islander	20 955 083 (6.4)	180 311 (2.2)	119 279 (2.0)
Black	43 319 362 (13.2)	1 383 616 (16.7)	1 087 871 (18.0)
Hispanic	60 404 487 (18.4)	596 537 (7.2)	430 393 (7.1)
White	200 897 980 (61.2)	6 045 752 (73.1)	4 348 260 (72.1)
Sex, No. (%)^d^			
Male	161 692 336 (49.2)	9 393 350 (85.9)	6 010 777 (87.9)
Female	166 637 617 (50.8)	1 230 007 (11.2)	618 829 (9.1)

Abbreviation: VA, US Department of Veterans Affairs.

^a^ The 3 populations are fully nested: VA active users are included within VA enrollees, which are included within the US population. The VA demographic data are for the start of fiscal year 2019, which ran from October 2018 to September 2019.

^b^ Data were missing for 430 740 enrollees and 278 985 active users.

^c^ The race and ethnicity categories are mutually exclusive. Data were missing for 2 672 036 enrollees and 808 309 active users.

^d^ Data were missing for 316 579 enrollees and 207 952 active users.

**Table 2.** Rates of Observed and Expected All-Cause Deaths, Rates of Excess Deaths, and Relative Increases, April to December 2020

Deaths	US population	VA population	
		Enrollees	Active users
**All-cause deaths/100 000** ^a^			
Expected 2020 Q2-4, No. (95% prediction interval)	930 (910-960)	2520 (2360-2670)	2910 (2770-3050)
Observed 2020 Q2-4, No.	1130	3022	3441
Excess incidence, No. (95% prediction interval)	200 (170-220)	500 (360-660)	530 (390-680)
Risk ratio (95% prediction interval)	1.22 (1.19-1.25)	1.20 (1.13-1.28)	1.18 (1.13-1.24)
Reported COVID-19 deaths/100 000	170	NA	NA
**Standardized deaths/100 000** ^a^ ^,^ ^b^			
Expected 2020 Q2-4, No. (95% prediction interval)	2160 (2130-2200)	2360 (2210-2500)	2730 (2590-2860)
Observed 2020 Q2-4, No.	2588	2842	3256
Excess incidence, No. (95% prediction interval)	430 (390-460)	480 (350-630)	530 (400-670)
Risk ratio (95% prediction interval)	1.20 (1.17-1.22)	1.20 (1.14-1.29)	1.19 (1.14-1.26)

Abbreviations: NA, not applicable; Q, quarter; VA, US Department of Veterans Affairs.

^a^ All mortality rates calculated as deaths per 100 000 people aged 25 years or older during Q2 to Q4 of 2020. Expected incidence and excess incidence are rounded to the nearest 10. The observed and standardized rates do not align perfectly for VA enrollees because of the exclusion of those with missing race and ethnicity from the standardization calculation. Expected incidence and risk ratios, along with the corresponding 95% prediction intervals, were derived from the regression model.

^b^ Direct standardization by age group, sex, race and ethnicity, and region, using the 2020 VA enrollee population as the reference population.

### Relative and Absolute Increases in Rates of Death in 2020

**Figure 1.**
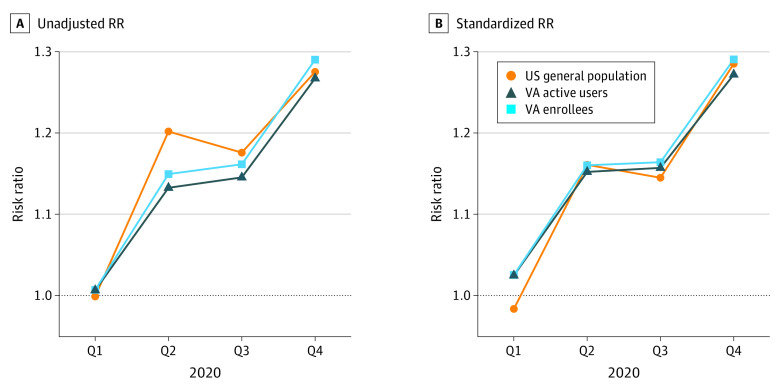
Trajectory of the Risk Ratio (RR) for the General US Population, Veterans Affairs (VA) Enrollees, and VA Active Users A, RRs calculated using the raw mortality rates. B, RRs calculated using the mortality rates standardized by age, sex, race and ethnicity, and region. The closer alignment for the standardized plot suggests that any differences in the unadjusted plot are associated with demographic differences. Q indicates quarter.

**Figure 2.**
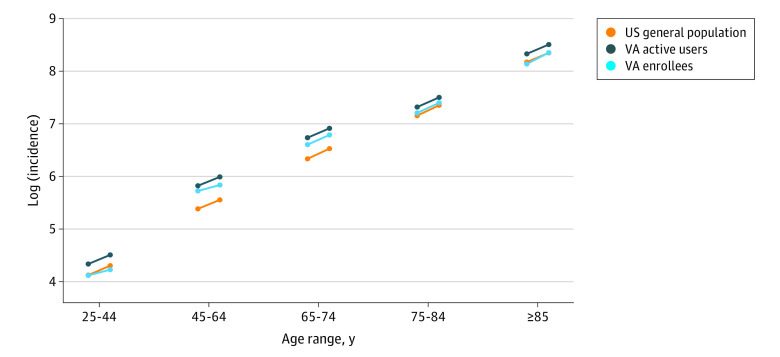
Observed Mortality Rates and Expected Mortality Rates in the General US Population, Veterans Affairs (VA) Enrollees, and VA Active Users, Stratified by Age Circles on the left sides of the lines are expected mortality rates, and circles on the right sides of the lines are observed mortality rates. The slope of the line indicates the relative increase during the pandemic (the risk ratio). Mortality rates are standardized based on the sex, race and ethnicity, region, and distribution of the 65- to 79-year VA enrollee population. Parallel slopes of the lines indicate a similar relative increase (risk ratio).

There were differences between the rates of observed and expected deaths due to any cause starting in the second quarter of 2020 (April onward). These increases were apparent across all of the adult age groups (≥25 years) in both the general US population and the VA populations (eFigure 1 in Supplement 1). There were subtle differences in the magnitude of the relative increases during the first wave of the pandemic (second quarter of 2020), with a larger increase in the general US population than in either of the VA populations (Figure 1A). However, these differences were largely due to differences in the geographic and demographic makeup of the populations, which were resolved by standardization (Figure 1B; eFigure 2 in Supplement 1). Across all of 2020, the relative increase in death rates during 2020 was similar in the general US population (RR, 1.20 [95% CI, 1.17-1.22]), among VA enrollees (RR, 1.20 [95% CI, 1.14-1.29]), and among VA active users (RR, 1.19 [95% CI, 1.14-1.26]). Because the prepandemic standardized mortality rates were higher in the VA populations prior to the pandemic, the absolute rates of excess mortality were higher in the VA population despite the similar relative increases in all 3 populations (Figure 1 and Figure 2; Table 2).

### Comparison of Excess Death Rates by Age, Sex, and Race and Ethnicity

The relative increase in deaths was smaller in the White population than in other racial and ethnic groups. These patterns were generally consistent between the general US population and the VA populations, but the disparities were less pronounced in the VA populations, particularly younger age groups (eFigure 3 in Supplement 1). In the general US population, the relative increase in death rates was similar between women and men (eFigures 4 and 5 in Supplement 1). Among VA active users, the relative increase was larger for men (eFigure 4 and 5 in Supplement 1). Among women and men, baseline death rates were higher for men, so the burden of the absolute increase was greater for men. Female VA enrollees had a large increase over baseline during the first quarter of 2020, which suggests that the baseline for this subgroup was not reliable (eFigure 5 in Supplement 1).

### Rates of Excess Deaths Compared With Recorded COVID-19 Deaths

Overall, in 2020, there were 200 excess deaths per 100 000 people (95% CI, 170-220 excess deaths per 100 000 people) in the US population. During the same period, there were 170 deaths per 100 000 people who were recorded as having COVID-19 as the underlying or associated cause (Table 2; eFigure 6 in Supplement 1). This finding suggests that approximately 85% of the increase in the death rate was directly associated with COVID-19 (170 COVID-19 deaths/200 total excess deaths). There were differences in the magnitude of the discrepancy between excess deaths and reported COVID-19 deaths by race and ethnicity (eFigure 6 in Supplement 1). This gap was larger among younger adults who were Black or American Indian or Alaska Native individuals (eFigure 6 in Supplement 1). Access to specific causes of death was not available for the VA population unless the individual received a test for SARS-CoV-2 within the VA system; therefore, similar comparisons between excess and reported deaths were not performed for the VA populations.

### Individual-Level Analysis

Estimates of the relative increase in death rates during 2020 among VA active users was similar between the Poisson model (RR, 1.18 [95% CI, 1.15-1.22]) and the patient-level Cox proportional hazards regression model (hazard ratio, 1.17 [95% CI, 1.15-1.20]) (eTable in Supplement 1). There was also no meaningful difference in the estimates by quarter.

## Discussion

In this study, we found that after adjusting for differences in age, sex, race and ethnicity, and region, the relative increase in rates of death during the first 9 months of the COVID-19 pandemic were similar between the general US population and enrollees in and users of the VA health care system. The absolute prepandemic death rate was higher in the VA populations, which translated to higher absolute excess death rates among veterans, despite the similar relative increases in the groups.

There are reasons to think that the outcomes for the VA active user population would have been more associated with the COVID-19 pandemic compared with the general US population. The VA population is heavily skewed toward men, has a high prevalence of comorbid conditions, and is enriched for those seeking medical care.^21^ All these factors can increase the risk of severe outcomes from COVID-19. The baseline death rate was higher in the VA populations, but the relative increase during the pandemic was not notably different. Our analysis demonstrated that the COVID-19 pandemic effectively acted as a multiplier on the baseline death rates. Future work could focus on estimating excess deaths with individual-level analyses, evaluating variations with comorbid conditions. Although this type of analysis would be challenging to do in the general US population due to a lack of electronic health records, the VA health system is well equipped to do so.

The increase in death rates during the pandemic differed by racial and ethnic group, which was consistent between the 3 populations. Previous work demonstrated that, once hospitalized, Black and White veterans had similar rates of death due to COVID-19.^22^ This finding suggests that differences in the association of the COVID-19 pandemic with outcomes are due to other factors, primarily the rate of infection in the different groups and rate of susceptibility to severe infection (eg, comorbidity). The risk of infection varied by race and ethnicity and region,^23^ and larger household size was also associated with greater infection risk.^24^

Measuring excess mortality has advantages and disadvantages as an analytical approach. By evaluating the overall increase in rates of death, regardless of cause, issues associated with variations in viral testing and differences in cause-of-death coding practices are mitigated. The downside is that there is not a perfect correlation between excess deaths and deaths caused by COVID-19. The magnitude of the excess deaths could result directly from deaths caused by COVID-19, or they could indirectly result from a number of factors, including avoidance of emergency care services, disruptions to routine or emergency care, changes in rates of other infectious diseases (eg, the disappearance of influenza in 2020), and changes in rates of violent crime and overdoses that might have been associated with pandemic disruptions.^2^ Our analyses show that the magnitude of the increase in death rates in the US was similar to the magnitude of recorded COVID-19 deaths (170 recorded COVID-19 deaths per 100 000 vs 200 overall excess deaths per 100 000). Modeling studies that make use of the time series of deaths support this finding and suggest that most of the increase in death rates among older adults was directly associated with the virus, based on the timing and trajectory of the increases in all-cause deaths.^2^ It is likely that many of the deaths in younger adult age groups were not directly associated with the virus, so other indirect factors, including worsening mental health and increases in substance abuse, are likely to have a larger relative association with deaths in younger age groups.^2^

### Limitations

These analyses have some important limitations. Race and ethnicity data were missing for 24% of VA enrollees and 7% to 14% of VA active users. The data for these individuals were effectively dropped when calculating the standardized rates. If the race and ethnicity data were missing at random, it would not introduce bias into the estimates. However, individuals with missing race and ethnicity information tend to be less dependent on VA health care and are more likely to be from racial and ethnic minority groups.^25^ Therefore, we likely undercounted the number of deaths in racial and ethnic groups during the pandemic. Although we were able to standardize comparisons by age, sex, race and ethnicity, and region, we did not have individual-level data on comorbidities in the general US population. Therefore, differences in the prevalence of comorbidities between veterans and the general US population that do not correlate with the adjusted factors could confound the comparisons.^26^ Estimates of excess deaths depend on the statistical modeling, including trend and seasonal components, being correctly specified. We used a multilevel model, which generally will generate a more stable model for sparse groups, but these types of models can also introduce bias for individual groups. This bias could lead to overestimation or underestimation of the trend for some groups, causing inaccurate estimates of excess deaths for subgroups. Future work could focus on further developing the individual-level models to account for additional indicators of underlying health status, which were not addressed here.

## Conclusions

Using detailed individual-level mortality records, this cohort study found that the relative association of the COVID-19 pandemic with all-cause mortality was similar between the general US population and users of the VA health care system. Future evaluations of excess deaths in different demographic and comorbidity groups in the general US and VA populations will help to contextualize these comparisons.

## Notes

Supplement 1. **eTable.** Comparison of Estimated Risk Ratio From Models Fit to Individual-Level Data or to Aggregated Data From VA Active Users **eFigure 1.** Time Series of Observed and Expected Rates Over Time by Age Group Among the General US Population and Among Enrollees of the VA **eFigure 2.** Standardized Risk Ratio by Quarter in 2020 (Observed/Expected), Stratified by Age and Region, Among White Men **eFigure 3.** Risk Ratio for Each Quarter in 2020 for (A) the General US Population and (B) VA Enrollees, and (C) VA Active Users, Calculated as Observed/Expected Deaths; Stratified by Age Group and Race/Ethnicity **eFigure 4.** Observed Mortality Rates (Open Symbols) and Expected Mortality Rates (Closed Symbols) in the US Population, VA Enrollees, and VA Active Users, Stratified by Age and Sex **eFigure 5.** Risk Ratio for Each Quarter in 2020 for (A) the General US Population and (B) VA Enrollees, and (C) VA Active Users, Calculated as Observed/Expected Deaths; Stratified by Sex **eFigure 6.** (A) Estimated Excess Deaths, (B) Deaths Recorded as Being Caused by COVID-19, and (C) the Difference Between These Two Quantities, by Quarter in 2020
eMethods.

eReference.

Supplement 2.
Data Sharing Statement
