# Supplementary material for: Excess Mortality Among Patients in the Veterans Affairs Health System Compared With the Overall US Population During the First Year of the COVID-19 Pandemic
Source: JAMA Netw Open. 2023 May 8;6(5):e2312140. doi: 10.1001/jamanetworkopen.2023.12140 (PMC10167568; doi:10.1001/jamanetworkopen.2023.12140)
Supplement: Supplement 1. — eTable. Comparison of Estimated Risk Ratio From Models Fit to Individual-Level Data or to Aggregated Data From VA Active Users eFigure 1. Time Series of Observed and Expected Rates Over Time by Age Group Among the General US Population and Among Enrollees of the VA eFigure 2. Standardized Risk Ratio by Quarter in 2020 (Observed/Expected), Stratified by Age and Region, Among White Men eFigure 3. Risk Ratio for Each Quarter in 2020 for (A) the General US Population and (B) VA Enrollees, and (C) VA Active Users, Calculated as Observed/Expected Deaths; Stratified by Age Group and Race/Ethnicity eFigure 4. Observed Mortality Rates (Open Symbols) and Expected Mortality Rates (Closed Symbols) in the US Population, VA Enrollees, and VA Active Users, Stratified by Age and Sex eFigure 5. Risk Ratio for Each Quarter in 2020 for (A) the General US Population and (B) VA Enrollees, and (C) VA Active Users, Calculated as Observed/Expected Deaths; Stratified by Sex eFigure 6. (A) Estimated Excess Deaths, (B) Deaths Recorded as Being Caused by COVID-19, and (C) the Difference Between These Two Quantities, by Quarter in 2020 eMethods. eReference. [file jamanetwopen-e2312140-s001.pdf]

Supplementary Online Content

Weinberger DM, Rose L, Rentsch C, et al. Excess mortality among patients in the Veterans Affairs health system compared with the overall US population during the first year of the COVID-19 pandemic. *JAMA Netw Open.* 2023;6(5):e2312140. doi:10.1001/jamanetworkopen.2023.12140

eTable. Comparison of Estimated Risk Ratio From Models Fit to Individual-Level Data or to Aggregated Data From VA Active Users
eFigure 1. Time Series of Observed and Expected Rates Over Time by Age Group Among the General US Population and Among Enrollees of the VA
eFigure 2. Standardized Risk Ratio by Quarter in 2020 (Observed/Expected), Stratified by Age and Region, Among White Men
eFigure 3. Risk Ratio for Each Quarter in 2020 for (A) the General US Population and (B) VA Enrollees, and (C) VA Active Users, Calculated as Observed/Expected Deaths; Stratified by Age Group and Race/Ethnicity
eFigure 4. Observed Mortality Rates (Open Symbols) and Expected Mortality Rates (Closed Symbols) in the US Population, VA Enrollees, and VA Active Users, Stratified by Age and Sex
eFigure 5. Risk Ratio for Each Quarter in 2020 for (A) the General US Population and (B) VA Enrollees, and (C) VA Active Users, Calculated as Observed/Expected Deaths; Stratified by Sex
eFigure 6. (A) Estimated Excess Deaths, (B) Deaths Recorded as Being Caused by COVID-19, and (C) the Difference Between These Two Quantities, by Quarter in 2020
eMethods.
eReference.

This supplementary material has been provided by the authors to give readers additional information about their work.

© 2023 Weinberger DM et al. *JAMA Network Open.*

eTable. Comparison of Estimated Risk Ratio From Models Fit to Individual-Level Data or to Aggregated Data From VA Active Users. The risk ratio quantifies changes in rates of death in 2020 (overall or by quarter) compared to 2018-2019. Both models adjusted for age group, sex, race/ethnicity, and region

Model type	IRR/HR Overall		IRR/HR 2020Q1	IRR/HR 2020Q2	IRR/HR 2020Q3	IRR/HR 2020Q4
Individual-level (Cox)	1.17 (1.15, 1.20)		1.02 (0.99, 1.05)	1.15 (1.12, 1.18)	1.19 (1.16, 1.22)	1.35 (1.32, 1.39)
Aggregated data (Poisson)	1.18 (1.15, 1.22)		1.00 (0.96, 1.04)	1.10 (1.06, 1.15)	1.15 (1.10, 1.20)	1.29 (1.24, 1.35)

© 2023 Weinberger DM et al. *JAMA Network Open.*

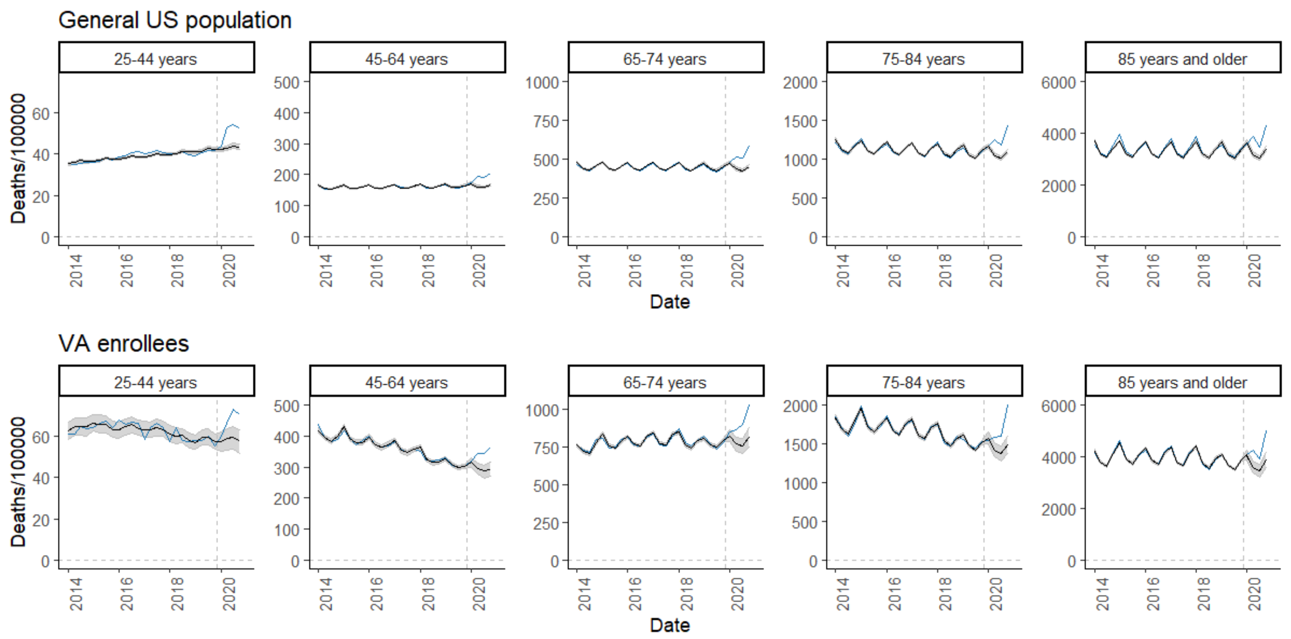

eFigure 1. Time Series of Observed and Expected Rates Over Time by Age Group Among the General US Population and Among Enrollees of the VA. The black line with the shaded area indicates the fitted values (up through 2019) and predicted values (for 2020), while the colored lines show the observed incidence by quarter. The divergence of the observed and expected rates gives the excess incidence.

© 2023 Weinberger DM et al. *JAMA Network Open.*

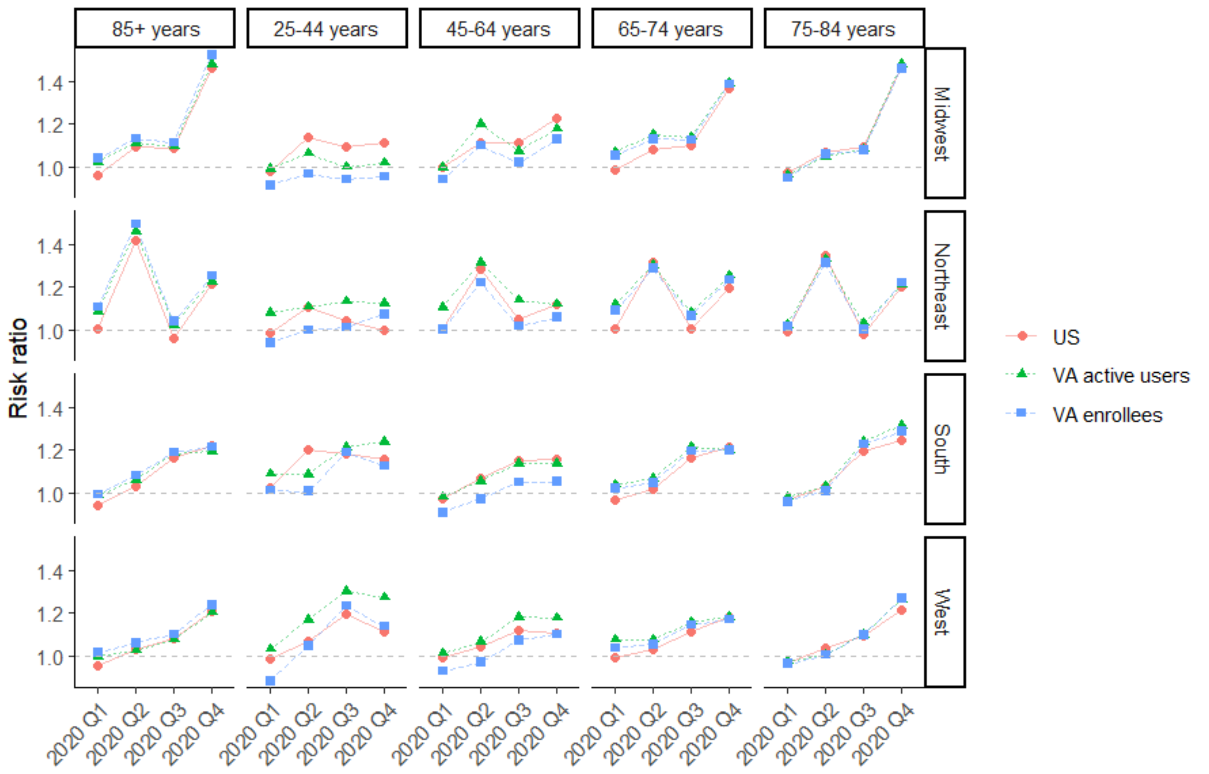

eFigure 2. Standardized Risk Ratio by Quarter in 2020 (Observed/Expected), Stratified by Age and Region, Among White Men

© 2023 Weinberger DM et al. *JAMA Network Open.*

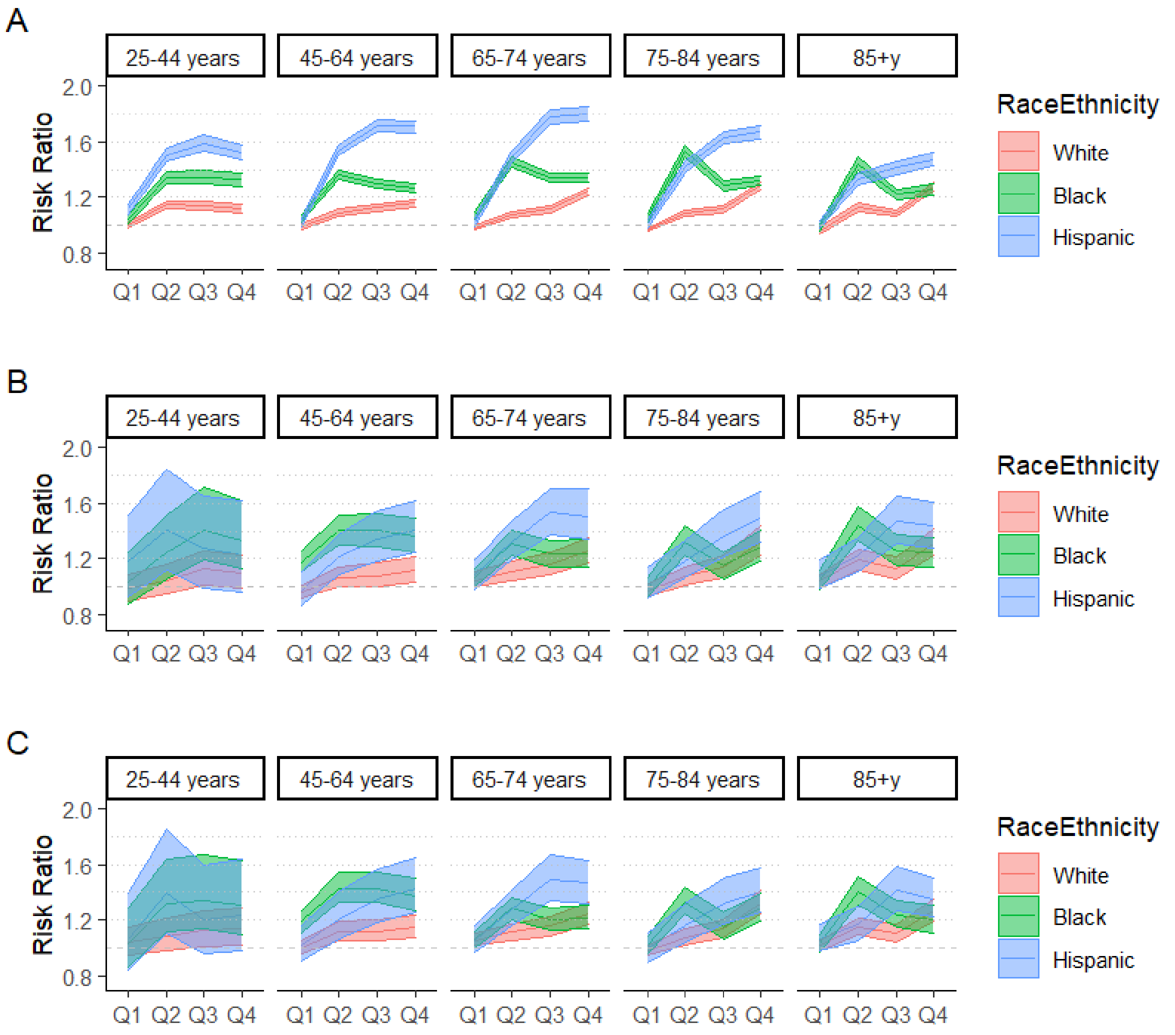

eFigure 3. Risk Ratio for Each Quarter in 2020 for (A) the General US Population and (B) VA Enrollees, and (C) VA Active Users, Calculated as Observed/Expected Deaths; Stratified by Age Group and Race/Ethnicity. Only White, Black, and Hispanic categories are shown due to higher degrees of uncertainty for the other strata.

© 2023 Weinberger DM et al. *JAMA Network Open.*

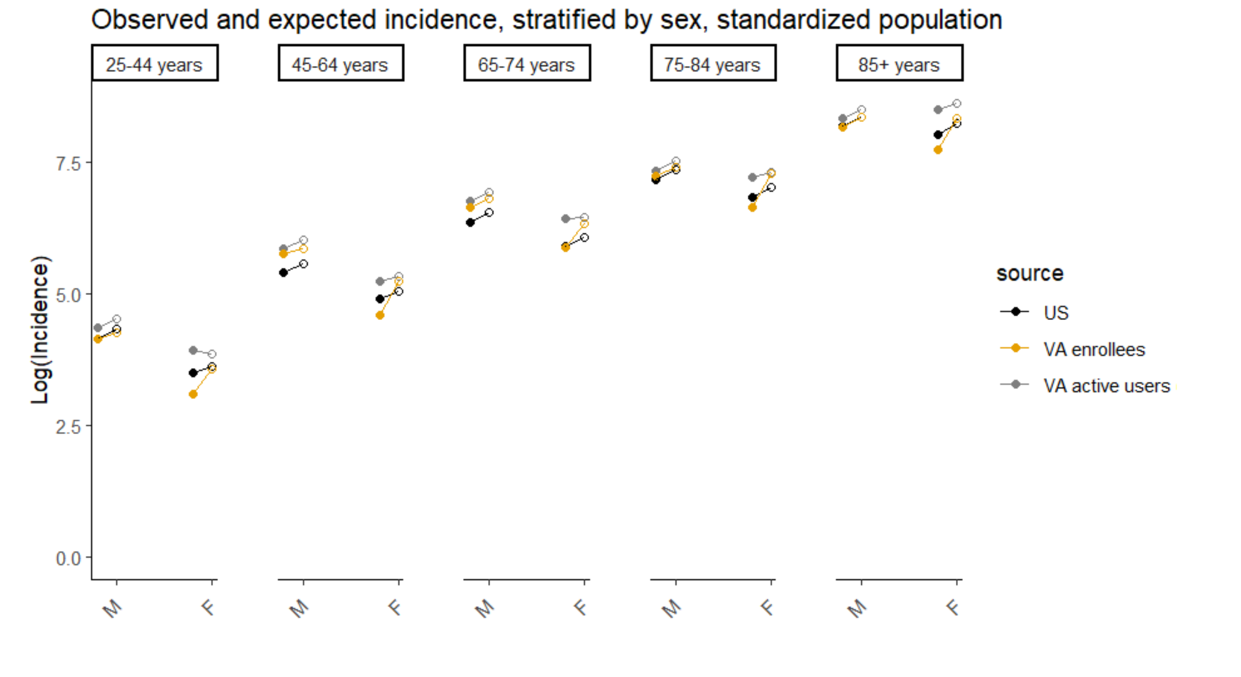

eFigure 4. Observed Mortality Rates (Open Symbols) and Expected Mortality Rates (Closed Symbols) in the US Population, VA Enrollees, and VA Active Users, Stratified by Age and Sex. Slope of the line indicates the relative increase during the pandemic. Mortality rates are standardized based on the region and race/ethnicity distribution of the 65-74 year old VA enrollee population

© 2023 Weinberger DM et al. *JAMA Network Open.*

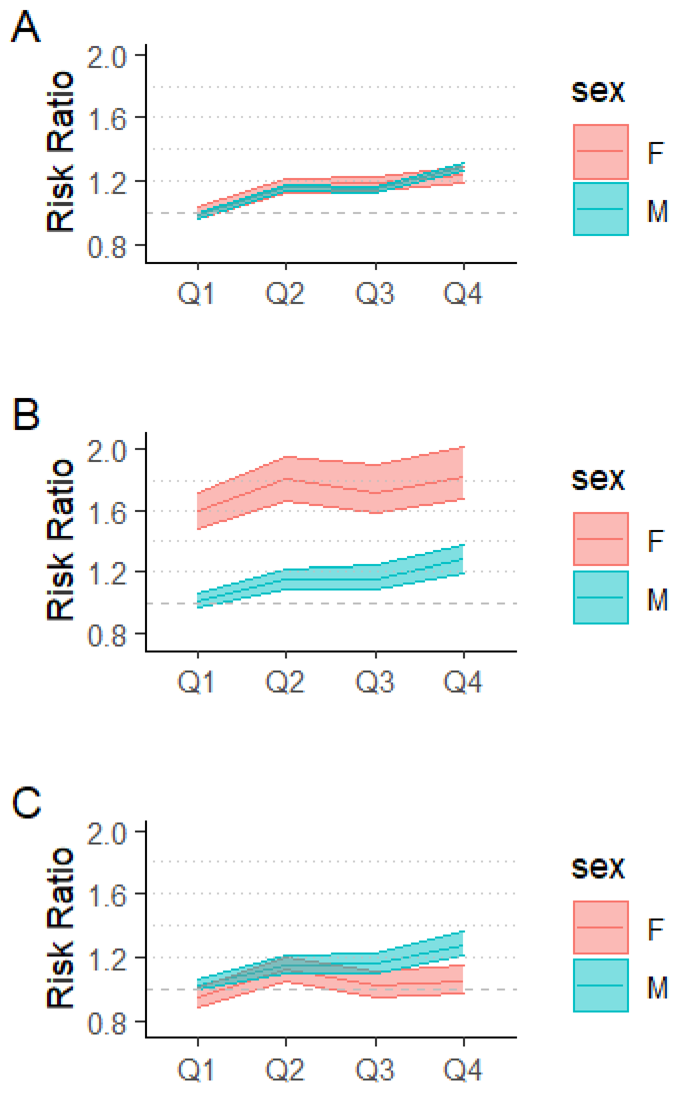

eFigure 5. Risk Ratio for Each Quarter in 2020 for (A) the General US Population and (B) VA Enrollees, and (C) VA Active Users, Calculated as Observed/Expected Deaths; Stratified by Sex

© 2023 Weinberger DM et al. *JAMA Network Open.*

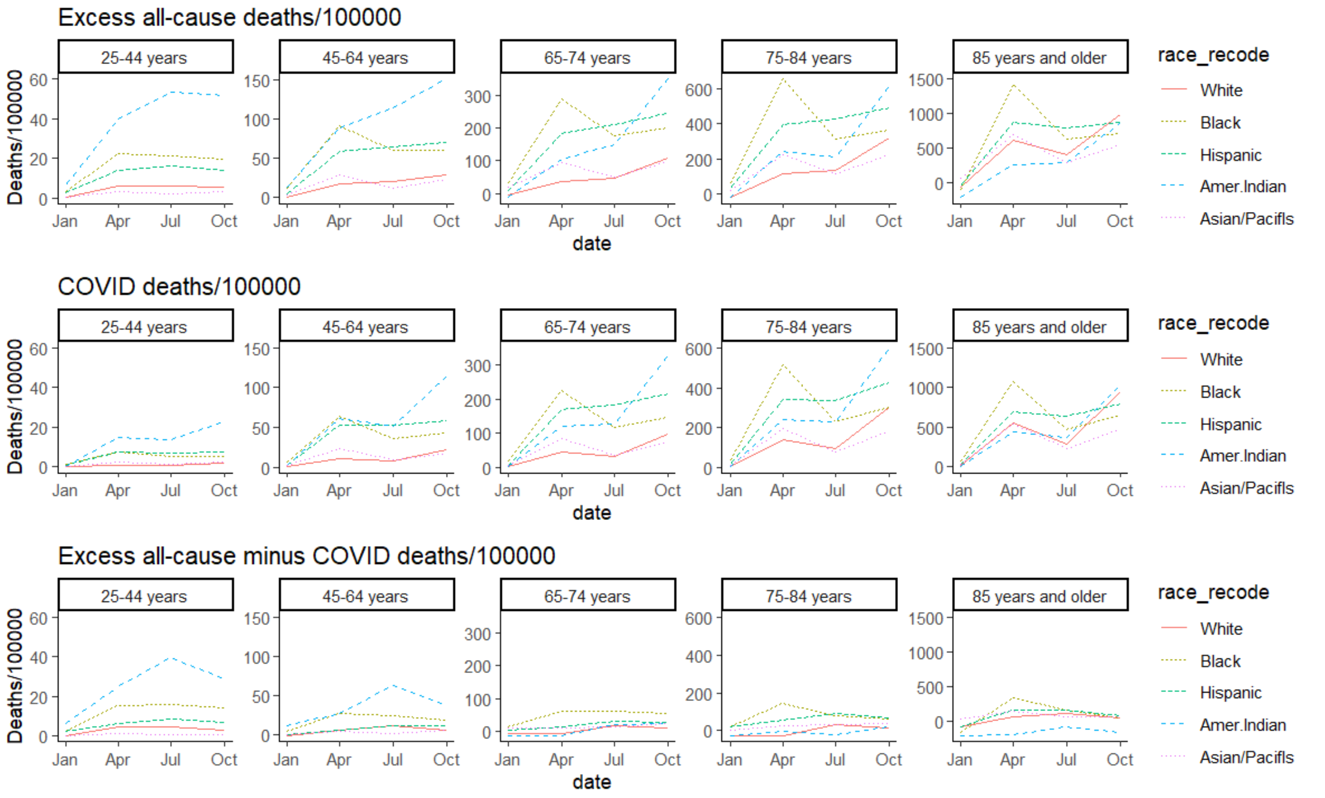

eFigure 6. (A) Estimated Excess Deaths, (B) Deaths Recorded as Being Caused by COVID-19, and (C) the Difference Between These Two Quantities, by Quarter in 2020. Separate plots are presented by age group, and are stratified by race/ethnicity (White, Black, Hispanic, American Indian, Asian/Pacific Islanders).

© 2023 Weinberger DM et al. *JAMA Network Open.*

eMethods.

Model details

Data in any given subgroup are sparse, making it difficult to fit separate models for each subgroup (as was done in previous work [1]). Instead, we used a multilevel Bayesian analysis approach. With this approach, we fit a model to data aggregated by time period, race/ethnicity, age group, region, and sex. Parameters were estimated in a multilevel modeling framework, so information was shared between categories of race/ethnicity, age group, region, and sex. The model adjusted for seasonality using categorical variables for quarter, adjusted for linear time trends (by quarter and year), and uses a population offset to adjust for the size of the population. An AR(1) random intercept was included to capture unexplained variability in the data that was common across the different categories of race/ethnicity, age group, region, and sex. The model for the number of deaths in age group *i*, race/ethnicity group *j*, sex *k*, region *r*, and time *t* was:

$$Y_{ijkrt} \sim \text{Poisson}(\text{lambda}_{ijkrt})$$

$$\log(\text{lambda}_{ijkrt}) = \boldsymbol{\alpha}_{ijkr} + \boldsymbol{\beta}_{1,ijk}*\text{time} + \boldsymbol{\beta}_{2,ijk}*\text{qtr2} + \boldsymbol{\beta}_{3,ijk}*\text{qtr3} + \boldsymbol{\beta}_{4,ijk}*\text{qtr4} + \boldsymbol{\phi}_t$$

The intercept for each subgroup is given as

$$\boldsymbol{\alpha}_{ijkr} = a_0 + b_{0,i} + c_{0,j} + d_{0,k} + \boldsymbol{\psi}_{ijkr}$$

The trend and effect of seasonality for each subgroup is given as

$$\text{For N}\{1:4\}: \boldsymbol{\beta}_{N,ijk} = a_N + b_{N,i} + c_{N,j} + d_{N,k}$$

$\boldsymbol{\phi}_t$ is an AR(1) random intercept, which captures unexplained temporal variation that is shared across the different subgroups. a_x, b_x, c_x, d_x represent the contributions to the intercept, slope and quarterly effects by age group (b), race/ethnicity group (c), and sex (d). A separate intercept ($\boldsymbol{\alpha}_{ijkr}$) was estimated for each region, but the time trends and seasonal effects ($\boldsymbol{\beta}_{N,ijk}$) were shared across regions. These models were fit using the INLA package in R, which uses approximate Bayesian inference. Samples from the joint posterior distribution were obtained using the inla.posterior.sample() function. Samples were combined across subgroups for calculating summary measures and uncertainty intervals.

The a_N are assigned weakly informative priors (N(0,1)); the b_N, c_N, and d_N have shrinkage priors (N(0, tau_m)) and use the 'Z-model' formulation for random effects in INLA. Models were fit separately to the time series for the different populations (e.g., US population, VA active users).

Individual-level analysis

© 2023 Weinberger DM et al. *JAMA Network Open.*

We leveraged the full patient-level data from VA active users to compare estimates of excess mortality using the Poisson model described above, which are fit to aggregated data, with a patient-level Cox proportional hazards model, fit to individual-level data (Supplementary Methods). To reduce computational burden, we limited the pre-pandemic time period in this post-hoc analysis to 2 years (i.e., 2018-2019) for both the individual-level and aggregate models, and a linear trend was not included due to the short baseline period. The Cox model was specified using the same variables as the Poisson models and used current age as the time-scale. The Cox model was:

$$h(a|X) = h_0(a) * \exp(\, \delta * \text{Pandemic} + \sum_k^3 \beta_k Qtr_k + \sum_j^5 \gamma_j Race_{Ethnicity_j} + \theta * Female + \sum_n^3 \mu_n Region_n + \sum_k^3 \epsilon_k * Pandemic * Qtr_k + \sum_j^5 \tau_j * \text{Pandemic} * Race\_Ethnicity_j + \varphi * \text{Pandemic} * Female + \sum_n^3 \omega_n * \text{Pandemic} * Region_n$$

Where:

- $h(a|X)$is the hazard function at age a, given the covariates X
- $h_0(a)$ is the baseline hazard function at a specific age
- The model included main effects for race/ethnicity, sex, quarter, region, and pandemic period; as well as interactions between pandemic period and each of the other effects

© 2023 Weinberger DM et al. *JAMA Network Open.*

eReference.

1. Weinberger DM, Chen J, Cohen T, et al. Estimation of excess deaths associated with the COVID-19 pandemic in the United States, March to May 2020. JAMA Intern Med. American Medical Association (AMA); **2020**; 180(10):1336–1344.

© 2023 Weinberger DM et al. *JAMA Network Open.*
