# Supplementary material for: Excess Mortality Among Patients in the Veterans Affairs Health System Compared With the Overall US Population During the First Year of the COVID-19 Pandemic
Source: JAMA Netw Open. 2023 May 8;6(5):e2312140. doi: 10.1001/jamanetworkopen.2023.12140 (PMC10167568; doi:10.1001/jamanetworkopen.2023.12140)
Supplement: Supplement 2. — Data Sharing Statement [file jamanetwopen-e2312140-s002.pdf]

Data Sharing Statement

Weinberger. Excess Mortality Among Patients in the Veterans Affairs Health System Compared With the Overall US Population During the First Year of the COVID-19 Pandemic. *JAMA Netw Open*. Published May 08, 2023. doi:10.1001/jamanetworkopen.2023.12140

Data
Data available: No

Additional Information
Explanation for why data not available: Data on US mortality, with the exception of state/region, can be obtained from https://www.cdc.gov/nchs/nvss/mortality_public_use_data.htm. For additional variables, including geography, a data use agreement with NCHS is required. VA data and the analytic data sets used for this study can be made available to researchers with a VA IRB approved study protocol and data use agreement. Information is available at https://www.virec.research.va.gov or contact the VA Information Resource Center at VIReC@va.gov.
